# DNA repair deficiency biomarkers and the 70-gene ultra-high risk signature as predictors of veliparib/carboplatin response in the I-SPY 2 breast cancer trial

**DOI:** 10.1038/s41523-017-0025-7

**Published:** 2017-08-25

**Authors:** Denise M. Wolf, Christina Yau, Ashish Sanil, Annuska Glas, Emanuel Petricoin, Julia Wulfkuhle, Tesa M. Severson, Sabine Linn, Lamorna Brown-Swigart, Gillian Hirst, Meredith Buxton, Angela DeMichele, Nola Hylton, Fraser Symmans, Doug Yee, Melissa Paoloni, Laura Esserman, Don Berry, Hope Rugo, Olufunmilayo Olopade, Laura van ‘t Veer

**Affiliations:** 1 Department of Laboratory Medicine, University of California, San Francisco, San Francisco, CA 94115 USA; 2 Department of Surgery, University of California, San Francisco, San Francisco, CA 94115 USA; 3 Berry Consultants, LLC, Austin, TX 78746 USA; 4 Agendia, Inc., 1098XH Amsterdam, The Netherlands; 5 Center for Applied Proteomics and Molecular Medicine, George Mason University, Fairfax, Virginia 22030 USA; 6 Division of Molecular Pathology, Netherlands Cancer Institute, Amsterdam, The Netherlands; 7 QuantumLeap Healthcare Collaborative, San Francisco, CA 94143 USA; 8 Division of Hematology Oncology, University of Pennsylvania, Philadelphia, PA 19104 USA; 9 Department of Radiology, University of California, San Francisco, San Francisco, CA 94115 USA; 10 Division of Pathology, University of Texas, MD Anderson, Houston, TX 77030 USA; 11 Department of Medicine, University of Minnesota, Minneapolis, MN 55455 USA; 12 Department of Medicine, University of California, San Francisco, San Francisco, CA 94115 USA; 13 Department of Medicine, University of Chicago, Chicago, IL 60637 USA

## Abstract

Veliparib combined with carboplatin (VC) was an experimental regimen evaluated in the biomarker-rich neoadjuvant I-SPY 2 trial for breast cancer. VC showed improved efficacy in the triple negative signature. However, not all triple negative patients achieved pathologic complete response and some HR+HER2− patients responded. Pre-specified analysis of five DNA repair deficiency biomarkers (BRCA1/2 germline mutation; PARPi-7, *BRCA1*ness, and CIN70 expression signatures; and PARP1 protein) was performed on 116 HER2− patients (VC: 72 and concurrent controls: 44). We also evaluated the 70-gene ultra-high risk signature (MP1/2), one of the biomarkers used to define subtype in the trial. We used logistic modeling to assess biomarker performance. Successful biomarkers were combined using a simple voting scheme to refine the ‘predicted sensitive’ group and Bayesian modeling used to estimate the pathologic complete response rates. BRCA1/2 germline mutation status associated with VC response, but its low prevalence precluded further evaluation. PARPi-7, *BRCA1*ness, and MP1/2 specifically associated with response in the VC arm but not the control arm. Neither CIN70 nor PARP1 protein specifically predicted VC response. When we combined the PARPi-7 and MP1/2 classifications, the 42% of triple negative patients who were PARPi7-high and MP2 had an estimated pCR rate of 75% in the VC arm. Only 11% of HR+/HER2− patients were PARPi7-high and MP2; but these patients were also more responsive to VC with estimated pathologic complete response rates of 41%. PARPi-7, *BRCA1*ness and MP1/2 signatures may help refine predictions of VC response, thereby improving patient care.

## Biomarkers: Gene expression tests predict response to PARP inhibitor combined with carboplatin

Several predictive gene signatures can help identify breast cancer patients likely to respond to veliparib, an investigational PARP inhibitor, combined with the chemotherapy agent carboplatin. A team led by Denise Wolf, Christina Yau, and Laura van ‘t Veer from the University of California, San Francisco, used data from the I-SPY 2 trial to assess the predictive value of six different biomarkers in determining which women with early stage and locally advanced, aggressive breast cancer would have no signs of disease after veliparib—carboplatin treatment. They found three biomarkers with predictive value: a 7-gene expression signature that predicts breast cancer cell line sensitivity to another PARP inhibitor called olaparib; a 77-gene expression signature that detects molecular features shared with BRCA1-mutant tumours; and a 70-gene signature of recurrence risk called MammaPrint.

## Introduction

Poly(ADP-ribose) polymerase (PARP) inhibitors belong to an emerging class of drugs that operate on the principle known as ‘synthetic lethality’.^1, 2^ PARP is involved in single strand break (SSB) DNA repair; and upon PARP inhibition, some SSBs are converted into double strand breaks (DSBs) at replication forks. In homologous repair competent cells, DSBs are repaired so that the cells can survive.^3^ However, in homologous repair-deficient cells, DSBs are repaired via the less accurate non-homologous end joining pathway or by single strand annealing, resulting in aberrant chromatids that trigger cell death.^4^ Thus, cells with BRCA mutations or other homologous repair defects^5–7^ are preferentially sensitive to PARP inhibitors.

The PARP inhibitor, olaparib, was recently FDA approved for BRCA1/2 mutation positive patients with advanced ovarian cancer. There are at least five PARP inhibitors under clinical investigation, including olaparib (AstraZeneca, London), veliparib (ABT-888; Abbott Laboratories, IL), rucaparib (AG014699; Clovis Oncology), niraparib (MK4827; Merck), and talazoparib (BMN673; Medivation, CA)^8–15^ for BRCA-associated, triple negative (TN) and/or basal breast cancers and other solid tumors, either as monotherapy in DNA repair deficient patients or as potentiating agents for radiation or DNA-damaging anticancer agents.^16–18^ Results from clinical trials in breast cancer in the advanced disease setting appear to vary depending on patient characteristics and the therapeutic regimen under investigation.^19^ Phase 2 trials showed that olaparib as monotherapy led to objective response rates in 31–41% of BRCA1/2 mutation carriers who had previously received several courses of chemotherapy.^14, 20, 21^ However, results for BRCA1/2 wild-type breast cancer patients have been inconsistent. TN breast cancer patients without BRCA mutations largely did not respond to olaparib monotherapy in a phase 2 trial,^22^ whereas preclinical studies and phase 1 trials suggested that PARP inhibitors can be efficacious in these patients when combined with DNA damaging cytotoxic agents.^17^

**Fig. 1:**
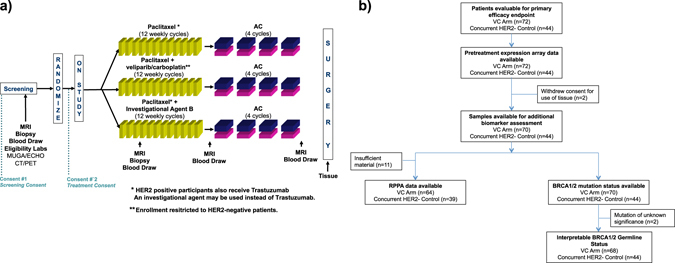
**a** I-SPY 2 TRIAL design schematic. Only patients with HER2− disease were eligible for randomization to the VC arm. **b** Consort diagram for the VC arm and HER2− concurrent controls, showing data availability for biomarker analysis

Veliparib in combination with carboplatin (VC) was recently evaluated in I-SPY 2, a multi-center Phase 2 adaptive standing platform trial for women with early stage, locally advanced, aggressive breast cancer.^23^ I-SPY 2 is designed to screen multiple experimental regimens in addition to standard neoadjuvant chemotherapy (Fig. 1a). Women with HER2+ disease also receive trastuzumab or other HER2-targeted therapy. This trial is adaptive, in that a patient randomized to receive experimental treatment is assigned preferentially to the arm where her cancer subtype is most likely to respond. Subtype is defined by hormone receptor (HR) status, HER2 status, and MammaPrint (MP) High 1/High 2 risk status (MP1/2), essentially a further stratification of the MP poor prognosis group (MP High) into high and ultra-high risk groups. The primary endpoint is pathologic complete response (pCR), i.e., no invasive cancer left in the breast or lymph nodes. The goal of I-SPY 2 is to identify (graduate) regimens with >85% predicted probability of succeeding in a 1:1 randomized 300-patient phase 3 trial where pCR is the endpoint, in the signatures defined by HR, HER2, and MP where the drug is most effective (graduates). The VC arm was open for enrollment to HER2-negative (HER2−) patients, and VC was eligible for graduation in three signatures: HER2-negative, HR-positive HER2-negative (HR+HER2−), and TN.

VC successfully graduated in the TN signature;^23^ but not all TN patients in the VC arm achieved a pCR, and many outside this subset responded. There is a need for additional robust biomarkers that predict VC sensitivity. Just as the I-SPY 2 standing platform trial is an efficient mechanism for screening promising agents for Phase 3 testing, the biomarker component of I-SPY 2, where samples are profiled for gene expression, protein levels and mutation status, provides a centralized resource for investigators to propose and test additional mechanism-of-action-based biomarkers using a rigorous, pre-defined analysis methodology in the context of the established clinically relevant biomarkers already incorporated within the trial. Previous studies have proposed homologous DNA repair deficiency (HRD) biomarkers, such as BRCA1/2 mutation,^24, 25^ BRCA1 promoter methylation,^26–28^ ‘BRCAness’ expression and CGH signatures,^27, 29^ Fanconi F and D methylation or loss,^30^ p53 and PTEN loss of function mutations or expression level,^31–33^ cell line based HRD/PARPi signatures,^34^ telomeric allelic imbalance,^35^ loss of heterozygocity HRD scoring,^36^ and HRD ‘genomic scarring’,^37^ among others,^27, 38, 39^ as possible markers of sensitivity to PARP inhibition. Through the I-SPY 2 Data access and Publication Committee process, five investigator-submitted qualifying biomarker concept proposals related to DNA damage repair deficiency were approved and individually evaluated in baseline (pre-treatment) biopsies as specific biomarkers of VC response.

The five proposed mechanism-of-action-based biomarkers were: (1) BRCA1/BRCA2 germline mutation; (2) a 7-gene DNA-repair deficiency expression signature (PARPi-7) that predicts breast cancer cell line sensitivity to the PARP inhibitor olaparib; ^34^ (3) a 77-gene *BRCA1*ness expression signature;^40, 41^ (4) the CIN70 chromosomal instability expression signature;^42, 43^ and (5) PARP1 protein levels, the target of veliparib. In addition, we also consider MP1/2 status, because the restricted enrollment of the VC arm to HER2− patients precluded the MP2 signature from being eligible for graduation. In this report, we present our evaluation of the predictive performance of each of these six biomarkers in the VC arm relative to the control arm, analyzed in isolation and in the context of the graduating TN subset. In addition, we compare these biomarkers to determine whether they are identifying the same patients, and perform an exploratory analysis that combines the most successful candidates into a composite VC sensitivity score.

## Results

### Patient characteristics summary

A total of 72 patients were randomized to the VC arm, and treated with veliparib and carboplatin in addition to standard taxane/anthracycline chemotherapy (VC+T−>AC). There were 44 concurrently randomized HER2− controls treated with standard chemotherapy (T−>AC).^23^ Patients who received non-protocol therapy, left their treating institution, or withdrew consent prior to surgery were considered non-pCR as per protocol. Patient and baseline clinical characteristics, such as age, ethnicity, HR status, tumor size and nodal status, were similar between the experimental and control arms.^23^ All 116 patients have Agilent 44 K expression arrays available. 98% of patients (*n* = 114) were evaluated for BRCA germline mutation status; and 89% (*n* = 103) have available RPPA data (Fig. 1b).

### BRCA1/2 germline mutation status

Of the 114 patients evaluated for BRCA1/2 germline mutation status, 15 were found to carry a deleterious or suspected deleterious BRCA1/2 mutation, with the majority (*n* = 11) occurring within the TN subtype. In the VC arm, BRCA mutation carriers were more likely to achieve a pCR compared to wild-type patients (75 vs. 29%) (Fig. S1). This translates to an odds ratio of 7.25 between the BRCA1/2 germline mutation carriers and wildtype patients (Fisher Exact test *p*-value = 0.006, Table 1). The small number of mutation carriers in the control arm (*n* = 3) precluded further evaluation.

**Table 1:** Associations between biomarkers and response within and between treatment arms

Biomarker	Platform	Type	Sample size		VC		Control		Biomarker x treatment interaction (Model A)		Biomarker x treatment interaction (Model B; adjusting for HR status)
			VC	Control	OR	P*	OR	P*	OR Ratio	LR p	LR p (adjusting for HR status)
BRCA1/2 Germline Mutation	Myriad BRCA Testing	Dichotomous	68	44	7.25	**0.006**	–	–	–	–	–
PARPi-7 Signature	Expression Array	Continuous	72	44	6.82	**0.0002**	0.99	0.98		**0.0018**	**0.0013**
BRCA1ness Signature	Expression Array	Dichotomous	72	44	3.2	**0.03**	0.39	0.45	2.14	**0.02**	**0.02**
CIN70 Signature	Expression Array	Dichotomous	72	44	3.12	0.052	0.97	1	1.19	0.24	0.22
MP1/2	Expression Array	Dichotomous	72	44	9.15	**<0.0001**	0.97	1	2.28	**0.03**	**0.03**
Total PARP protein	RPPA	Continuous	64	39	0.83	0.49	2.41	0.11		0.06	**0.04**
Cleaved PARP protein	RPPA	Continuous	64	39	1.17	0.56	0.46	0.36		0.2	0.11

Numbers in bold denote *p*<0.05

Model A (Logistic Regression Model):

pCR ~ Biomarker + Treatment + Biomarker × Treatment

Model B (Logistic Regression Model):

pCR ~ Biomarker + Treatment + Biomarker × Treatment + HR + HER2

* For dichotomous biomarkers, *p* value from a Fisher Exact test of the contingency table of pCR by biomarker status within each treatment arm is reported. For continous biomarkers, *p* value from a Ward test of a logistic regression model of pCR on biomarker levels within each treatment arm is reported

### The PARPi-7 and *BRCA1*ness signatures and MP1/2 classification specifically predict VC response

**Fig. 2:**
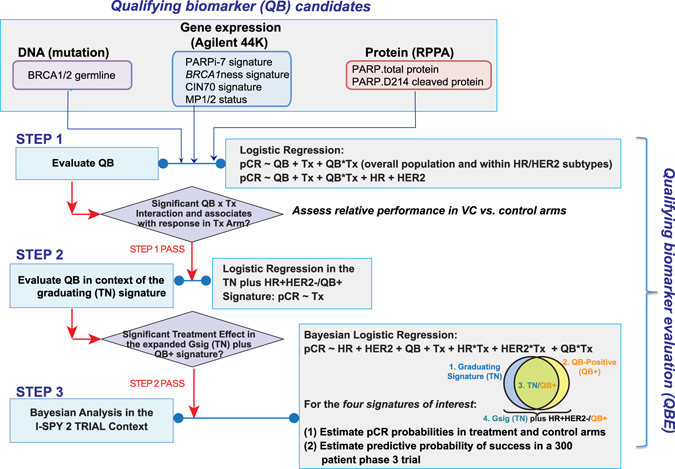
Biomarker analysis approach. Qualifying biomarker candidates are evaluated as specific predictors of response to VC using a predefined 3-step Qualifying Biomarker Evaluation (QBE) methodology, as shown in this flow diagram

We evaluated as proposed three dichotomous expression based signatures—the BRCA1ness signature, the CIN70 signature and the MP1/2 risk classification—and one continuous signature (PARPi-7) as qualifying biomarkers of VC response using our predefined qualifying biomarker evaluation (QBE) process (see Fig. 2).

The PARPi-7 and *BRCA1*ness signatures and the MP1/2 risk classification associated with response in the VC arm (*p* < 0.05), but not the control arm (Table 1). Using logistic regression, for each of these three signatures, the biomarker x treatment interaction term reflecting the relative biomarker performance between arms had a likelihood ratio (LR) test *p* value of <0.05, which remains significant (LR *p* < 0.05) upon adjusting for receptor subtype (Table 1).

Neither the CIN70 signature nor the total and cleaved PARP protein levels succeeded as specific biomarkers of response to VC (Table 1 and Fig. S2).

**Fig. 3:**
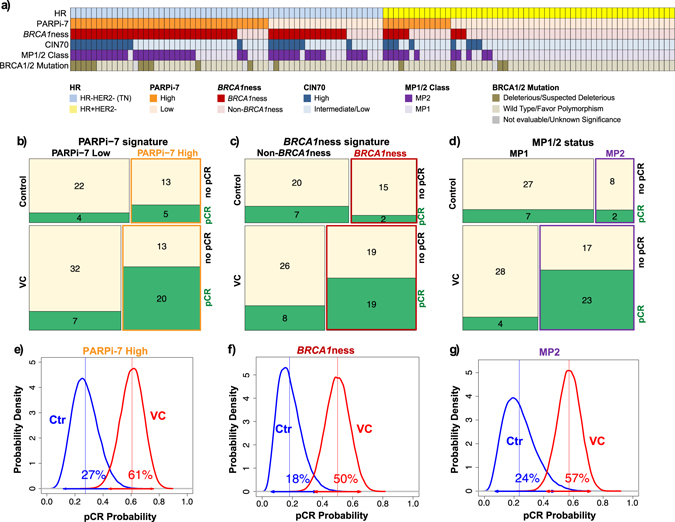
Qualifying biomarker performance. **a** Ordered heatmap showing the prevalence of all dichotomized biomarkers evaluated in this study, stratified by HR status. **b**–**d** Mosaic plots showing patient response stratified by treatment arm and **b** PARPi-7, **c** *BRCA1*ness (this figure panel is also jointly published in ref. [41]), and **d** MP1/2 signatures, respectively. **e**–**g** Bayesian estimated pCR probability distributions by treatment arm, for **e** PARPi7-high, **f** *BRCA1*ness, and **g** MP2 patients

For potential clinical utility and ease of comparison to the other successful signatures, we dichotomize the patients using the PARPi-7 signature at the published threshold (0.0372)^34^ [see Fig. S3] and use the dichotomous version of this signature henceforth. Figure 3a shows the biomarker signature prevalence and composition in the VC and control arms. pCR rates by receptor subtype, treatment arm, and biomarker are shown in Fig. S4. As shown in the mosaic plots in Fig. 3b–d, PARPi-7 High, *BRCA1*ness and MP2 patients all had a higher pCR rate in the VC arm (Fisher’s exact test *p* < 0.05), but not the control arm (*p* > 0.05), when compared to biomarker-negative patients.

We used Bayesian modeling, similar to the I-SPY 2 primary efficacy analysis, to estimate the pCR probabilities to VC and standard chemotherapy in biomarker predicted-sensitive patient subsets (PARPi-7 High, BRCA-like, and MP2). As shown in Fig. 3e–g, estimated pCR probabilities in the VC arm range from 50–61% vs. 18–27% in the control arm, in these subsets. We also computed the predictive probability of VC demonstrating superiority to control in a 1:1 randomized neoadjuvant phase 3 trial of 300 biomarker predicted-sensitive patients. These probabilities are >90% (95% for PARPi-7 High, 96% for *BRCA1*ness and 94% for MP2 patients). If we exclude the 15 BRCA1/2 germline mutation carriers from the analysis and consider only BRCA1/2 wild-type patients, these probabilities are 75% for PARPi-7 High, 81% for *BRCA1*ness and 87% for MP2 patients.

### Expanding the ‘predicted-responder’ group beyond TN using qualifying biomarkers

We evaluated the PARPi-7, *BRCA1*ness and MP1/2 signatures in relation to the graduating TN signature, by adding biomarker-positive HR+/HER2− patients to the TN group and evaluating whether the treatment effect of VC remains significant in this expanded population of ‘predicted sensitive’ patients. For all three signatures, the VC treatment effect in the expanded population remains similar to that observed within the graduating TN signature (see Table S2 for details); and the predicted probability of success in phase 3 in these expanded groups are comparable to the graduating signature (Fig. S5). For instance, if the PARPi-7 is used to expand the population by adding PARPi-7 High HR+HER2− patients to the TN group, the probability of VC demonstrating superiority to control in phase 3 is 90%, while increasing the prevalence of ‘predicted sensitive’ patients by 23% of HR+/HER2− patients in the trial. Similar results were observed when the *BRCA1*ness and MP1/2 signatures were considered (Fig. S5).

### Comparing and combining qualifying biomarkers in the context of HR and HER2 status to improve response prediction

Since PARPi-7, *BRCA1*ness, and MP1/2 all appear to succeed as specific predictors of VC combination therapy in I-SPY 2, we wondered whether these signatures identify the same patients, and if not, whether individual biomarkers could be combined to better identify likely responders within the TN and HR+/HER2− subsets. As shown in Fig. 3a, the concordance between biomarker pairs over all patients in the trial ranges from 66–78%, indicating that these biomarkers are not identifying exactly the same patients.

**Fig. 4:**
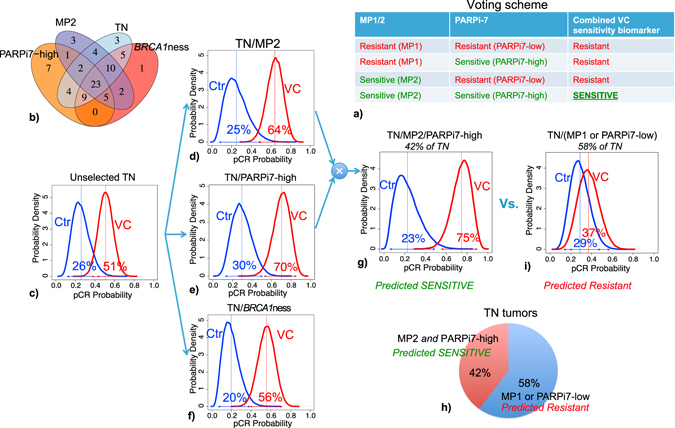
Combining VC-sensitivity markers in TN patients. **a** Simple voting scheme for combining biomarkers to refine sensitivity prediction. **b** Venn diagram showing overlap between VC-sensitivity biomarkers, including the graduating TN signature. **c–f** Bayesian estimated pCR probability distributions by treatment arm, for **c** unselected triple negative [TN], **d** TN/MP2, **e** TN/PARPi7-high, and **f** TN/BRCA1ness patients, respectively. **g**, **h** Bayesian estimated pCR probability distributions by treatment arm, for **g** predicted sensitive (TN/MP2/PARPi7-high) and **h** predicted resistant (TN/(MP1 or PARPi7-low)) triple negative patients. **i** Pie chart showing relative proportion of predicted sensitive vs. resistant TN patients

In our exploratory analysis that was not pre-specified, we combined pairs of biomarkers using a simple voting scheme. As shown in Fig. 4a, if patients are classified as resistant by both biomarkers, or resistant by one biomarker, they are classified as ‘resistant’. Patients are classified as ‘sensitive’ only if both biomarkers are in the ‘sensitive’ state.

#### Biomarker combinations within the TN subtype

Though 95% (57/60) of TN patients are PARPi7-high or MP2 or *BRCA1*ness, only 38% would be classified as sensitive by all three biomarkers (Fig. 4b).

Figure 4c shows the pCR probability distributions of TN patients in the VC and control arms, with estimated pCR rates of 51% in the VC arm and 26% in the control arm. In TN patients predicted VC-sensitive by a single additional biomarker, there is a greater separation in the pCR probability distributions between arms, with estimated pCR rates to VC ranging from 56% to 70% (Fig. 4d–f). If we combine the two biomarkers yielding the highest estimated pCR rate in the VC arm (PARPi-7 and MP2) to select TN patients who are TN/MP2/PARPi7-high (Fig. 4g), the separation of the estimated pCR rates between arms is greater still (75% in VC vs. 23% in control), with a predictive probability of success in phase 3 of 99% (Table S3). This group constitutes a sizeable 42% of TN patients in the trial (Fig. 4h). In contrast, the pCR probability distributions of TN patients with one or more markers in the resistant state (TN/PARPi7-low or TN/MP1) are nearly overlapping (Fig. 4i).

If we exclude the BRCA1/2 germline mutation carriers from the analysis, the separation of estimated pCR rates between arms is still large in the TN/MP2/PARPi7-high group (63% in VC vs. 23% in control), with a predictive probability of success in phase 3 of 95% (Fig. S6).

#### Biomarker combinations within the HR+/HER2− subtype

**Fig. 5:**
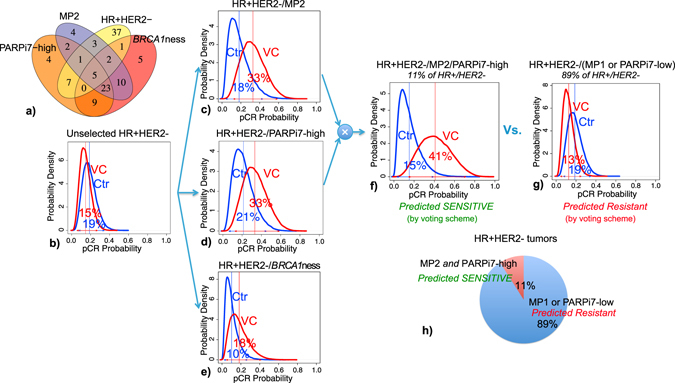
Combining VC-sensitivity markers in HR+HER2− patients. **a** Venn diagram showing overlap between VC-sensitivity biomarkers in the HR+HER2− subset. **b**–**g** Bayesian estimated pCR probability distributions by treatment arm, for **b** unselected HR+HER2−, **c** HR+HER2−/MP2, **d** HR+HER2−/PARPi7-high, and **e** HR+HER2−/BRCA1ness patients, respectively, and for HR+HER2− patients who are **f** predicted sensitive (HR+HER2−/MP2/PARPi7-high) and **g** predicted resistant (HR+HER2−/(MP1 or PARPi7-low)) by our simple voting scheme. **h** Pie chart showing relative proportion of predicted sensitive vs. resistant HR+HER2− patients

Only 34% (19/56) of HR+/HER2− patients are positive for one or more VC sensitivity biomarkers. 23% (13/56) are PARPi7-high, 20% (11/56) are MP2, and 14% (8/56) are *BRCA1*ness (Fig. 5a).

HR+/HER2− patients had low estimated pCR rates in both arms with nearly overlapping probability distributions (estimated pCR: 15% in VC, 19% in control; Fig. 5b). In HR+HER2− patients predicted VC sensitive by a single additional biomarker, there is a greater separation in the pCR probability distributions between arms with estimated pCR rates to VC of 33% in the HR+HER2−/MP2 and HR+HER2−/PARPi7-High subsets (Fig. 5c–e). If we combine MP1/2 and PARPi-7 per above to select HR+HER2− patients who are HR+HER2-/MP2/PARPi7-high (Fig. 5f), the separation of the estimated pCR rates between arms increases further (41% in VC vs. 15% in control), with a predictive probability of success in phase 3 of 82% (Fig. 5f; Table S3). In contrast, HR+/HER2− patients negative for at least one VC sensitivity marker (HR+HER2−/PARPi7-low, or HR+HER2-/MP1) have nearly overlapping pCR probability distributions with estimated pCR rates of 13% in VC vs. 19% in controls (Fig. 5g).

Although our results demonstrate that ‘double biomarker-positive’ in both TN and HR+HER2− patients may be more sensitive to VC than patients with fewer markers in the ‘sensitive’ state, the prevalence of MP2/PARPi7-high patients in the TN subset is relatively high (42%; Fig. 4i), whereas HR+/HER2− patients with these markers are relatively rare (11%; Fig. 5h).

## Discussion

The I-SPY 2 TRIAL design presents both opportunities and challenges for evidence-based biomarker testing. On one hand, the biomarker-rich nature of the trial, in which pre-treatment tumor biopsies are assayed on Agilent 44 K expression and RPPA protein/phospho-protein platforms, provides an excellent resource to investigate the molecular correlates of response and resistance. As well, collection of serial biopsies and blood samples over the course of therapy generates an invaluable repository for additional molecular profiling.

On the other hand, as the I-SPY 2 TRIAL was designed to efficiently evaluate multiple novel regimens compared to a shared control arm for future Phase 3 testing, the sample sizes available for our biomarker studies are small. As well, VC is given as a combination therapy within the I-SPY 2 trial. While assessing the relative performance of a biomarker in the experimental vs. control arms enables us to identify specific predictors of response to VC, it is impossible to assess whether the successful biomarkers described in our report are predictive of response to the individual agents within the combination. In addition, I-SPY 2 uses adaptive randomization within HR/HER2/MP defined subtypes to enable efficient matching of novel regimens with their most responsive clinically relevant signatures. This may result in the unbalanced prevalence of biomarker-positive subsets in the experimental and control arms if the biomarker of interest is correlated with a HR/HER2/MP subtype that is preferentially enriched or depleted in the experimental arm by the randomization engine. As well, there are multiple genes in each signature measured on several platforms, creating the problem of multiplicity, which is compounded by the evaluation of multiple proposals. Altogether, these challenges limit our ability to draw definitive conclusions. Thus, our statistics are descriptive rather than inferential; and our findings require further validation in future trials.

Four of the five evidence-based, mechanism-of-action related (qualifying) biomarkers included in this report are markers of DNA repair deficiency because of the large body of in vitro and in vivo evidence that DNA repair deficient cancers selectively respond to DNA damaging agents potentiated by PARP inhibition. Many studies support BRCA1/2 germline mutation status as the ‘gold standard’ biomarker for predicting sensitivity to PARP inhibitors as monotherapy. In I-SPY 2, BRCA1/2 mutation carriers were indeed more likely to respond to VC than BRCA1/2 wildtype patients, but the mutation prevalence was too low for a comparison to response in the control arm.

Of the three DNA repair based expression signatures we evaluated, two (PARPi7 and *BRCA1*ness) were associated with response in the VC arm, but not in the control arm, and had a significant biomarker x treatment interaction that retained significance upon adjusting for HR status. These two successful qualifying biomarker signatures were derived in very different ways. The PARPi-7 signature was the result of an effort to build a predictor of response to a different PARP inhibitor, olaparib, using a breast cancer cell line panel and a candidate gene set restricted to DNA repair genes in one of the six major repair pathways. The *BRCA1*ness signature, on the other hand, was developed as a classifier to identify cancers exhibiting a BRCA1-mutated copy number profile. Indeed, 80% (12/15) of BRCA+ patients were classified as *BRCA1*ness, along with many non-mutation carriers. Interestingly, the concordance between these signatures is only moderate, and while both *BRCA1*ness and PARPi7 have predictive signal, the nature of this signal differs. In the TN subset, the differential response in the VC and control arms in *BRCA1*ness cancers mostly manifests as a depletion of responders in the control arm rather than an increase in responders in the VC arm relative to unselected TNs. Whereas the difference in estimated pCR rates in VC vs. control for PARPi7-high patients is driven by a higher response rate in VC.

Neither the PARP1 protein levels nor the dichotomized CIN70 signature specifically predict response to VC. The lack of association observed between PARP1 protein levels and differential response has been seen elsewhere in PARP inhibitor trials, perhaps due to saturation properties of the antibody or to a disconnect between DNA repair deficiency and PARP1 levels as measured. For CIN70, the lack of association with response may be due to the small size of the population or a non-ideal quantile cutoff for dichotomizing the I-SPY 2 patients, as it associates with response as a continuous variable.

We did not test non-expression based HRD biomarkers found to associate with response to platinum-based DNA damaging agents, including the HRD-LOH/HRD-LST score or Myriad’s HRD test quantifying the level of ‘DNA scarring’ on tumor genomes.^36, 37^ As these measures are in a sense closer to a direct read-out of DNA repair deficiency, it may be interesting to examine their correlation to the expression-based biomarkers we evaluated and to compare their predictive performance in I-SPY 2. But tissue availability is limited; and careful consideration is needed in determining the optimal use of samples within the I-SPY 2 tissue repository.

Our analysis suggests that had HER2−/MP2 or TN/MP2 signatures been eligible for graduation of VC from the I-SPY 2 trial, they would have graduated along with the TN signature (with >85% predictive probability of success in phase 3). Though not explicitly developed as a DNA repair deficiency biomarker, preliminary analysis of pathway differences between MP1 and MP2 class tumors suggest that MP2 cancers have higher expression of cell cycle genes and DNA repair pathways other than homologous repair.^44^ This may explain why MP1/2 and PARPi-7 are not highly concordant, as the latter largely reflects HRD.

The biomarker component of I-SPY 2 must function in the context of I-SPY 2’s innovative adaptive trial design. VC graduated with a >85% probability of success in a future phase 3 trial, conducted not in the general breast cancer population, but in the TN subset. Thus, TN (HR−/HER2−) status is already a successful biomarker for VC, and we must try to understand how the novel mechanism-of-action based biomarkers we are testing add value to the graduating TN signature. To this end, we imagined two different purposes for a biomarker.

One purpose of a biomarker concerns clinical decision making for individual patients, also known as personalized oncology. For this application, the objective is to use all the available information to predict the likelihood of an individual patient’s response to a set of treatment options. For this type of analysis we combine successful biomarkers, such as the qualifying biomarkers and the graduating signature to refine the sensitivity prediction. For instance, we combine TN status with the PARPi-7 signature, to show that TN patients who are also PARPi7-high have a 70% probability of achieving pCR in the VC arm compared to 30% in the control arm. We also show how a simple voting method can be used to combine information from multiple sensitivity biomarkers (PARPi7 and MP2) to improve VC response prediction to 75% pCR probability. Moreover, MP2/PARPi7-high remains a strong predictor of response to VC relative to control in TN patients who are BRCA1/2 wild-type. This information would be useful for a patient selecting a treatment protocol, but may not be appropriate for patient selection in a trial because the improved performance of the biomarker comes at the expense of excluding a large segment of the TN population.

Another purpose is to use the novel biomarker together with the graduating group to improve patient selection in a future phase 3 trial of VC. In this setting, an ideal biomarker is one which identifies the maximum number of patients likely to have a sufficiently improved response to VC (relative to control) so as to succeed in a subsequent phase 3 trial conducted in this ‘biomarker-positive’ group. Maximizing the size of this biomarker-positive subset is desirable because phase 3 testing and thus approval of VC will likely be limited to this group. When we added single biomarker positive HR+HER2− patients to the graduating TN group, the Bayesian probabilities of success in phase 3 of this expanded biomarker-positive population is similar to the graduating TN signature, while increasing prevalence by adding 14–23%. Using the voting method, only 11% of HR+HER2− patients would be added to the predicted responder group. This example illustrates a generic trade-off between biomarker performance and prevalence (or specificity and sensitivity), with ‘fit for purpose’ implications for how these biomarkers are best used combinatorially.

Our future plans include evaluating whether the biomarkers predictive of VC response in the I-SPY 2 TRIAL can also predict sensitivity to other PARP inhibitors combined with DNA damaging agents or even carboplatin alone. In addition, we plan to use these biomarkers as part of a SMART-trial design to adapt treatment within patients who are not responding to their initial therapy.

## Methods

In the I-SPY 2 TRIAL (NCT01042379), HER2− patients were randomized to receive standard chemotherapy (paclitaxel followed by doxorubicin/cyclophosphamide; T−>AC) or the oral PARP inhibitor veliparib in combination with carboplatin and chemotherapy (VC+T−>AC).^23^ Pre-treatment samples from patients in the veliparib/carboplatin (VC) and concurrent control arms of the I-SPY 2 trial^23^ were profiled using Agilent 44 K expression arrays (Agendia, Inc) and reverse phase protein arrays (RPPA). Details of the sample preparation and data processing are provided in the Supplemental Methods. In addition, BRCA1/2 mutation status was assessed (Myriad Genetics Laboratories).

We evaluated six biomarkers as specific predictors of VC response using a pre-specified QBE methodology. Briefly, we follow a three-step methodology, first evaluating the relative performance of the biomarker between arms, followed by assessing biomarker performance in the context of the graduating (TN) signature. Finally, we perform Bayesian analysis to estimate pCR rates in the arms and the predictive probability of VC showing superiority to control in biomarker defined subsets. The six biomarkers evaluated are: (1) BRCA germline mutation; (2) a 7-gene DNA-repair deficiency expression signature (PARPi-7)^34^; (3) a 77-gene *BRCA1*ness expression signature;^40, 41^ (4) the CIN70 chromosomal instability expression signature;^42, 43^ (5) PARP1 protein levels; and (6) MP1/2 status. Details of the definition and scoring of each biomarker and our evaluation methodology are available in the Supplemental Methods. Each individual biomarker was evaluated separately; and these analyses did not account for multiplicities outside of the study.

In an exploratory analysis, we evaluate the concordance between successful biomarkers using the Kappa statistic. We use a simple voting method to combine the two most successful VC-sensitivity biomarkers and use Bayesian modeling to estimate the pCR rates and predictive probability of phase 3 trial success of biomarker-positive TN and HR+HER2− patients.

### I-SPY 2 TRIAL investigators

I-SPY 2 TRIAL Investigators, by site/affiliation: Avera Cancer Institute: Leyland-Jones B; British Colombia Cancer Agency: Chia S, Serpanchy R, Yu C; Emory University: McMillan S, Mosley R, Nguyen K, Wood EC, Zelnak A; Georgetown University: Dillis C, Donnelly R, Harrington T, Isaacs C, Kallakury B, Liu M, Lynce F, Oppong B, Pohlmann P, Tousimis E, Warren R, Willey S, Wong JE, Zeck J; Loyola University Chicago Medical Center: Albain K, Bartolotta MB, Bova D, Brooks C, Busby B, Czaplicki K, Duan X, Gamez R, Ganesh K, Gaynor E, Godellas C, Grace-Louthen C, Kuritza T, Lo S, Nagamine A, Perez C, Robinson P, Rosi D, Vaince F, Ward K; Innova Fairfax Hospital: Choquette K, Edmiston K, Gallimore H, McGovern J, Mokarem K, Pajaniappan M, Rassulova S, Scott K, Sherwood K, Wright J; Mayo Clinic, Arizona: Anderson KS, Gray RJ, Myers SJ, Northfelt DE, Pockaj BA, Roedig J, Wasif N; Mayo Clinic, Rochester: Arens AM, Boughey JC, Brandt KR, Carroll JL, Chen B, Connors AL, Degnim AC, Farley DR, Greenlee SM, Haddad TC, Hieken TJ, Hobday TJ, Jakub JW, Liberte LL, Liu MC, Loprinzi CL, Menard LR, Moe MM, Moynihan TJ, O’Sullivan C, Olson EA, Peethambaram PP, Ruddy KJ, Russell BA, Rynearson AL, Smith DR, Visscher DW, Windish AJ; H. Lee Moffitt Cancer Center and Research Institute: Cox K, Dawson K, Newton O, Ramirez W; Oregon Health and Science University: Bengtson H, Bucher J, Chui S, Gilbert-Ghormley B, Hampton R, Kemmer KA, Kurdyla D, Nauman D, Spear J, Wilson A; Swedish Cancer Institute: Beatty D, Dawson P, Ellis ER, Fer M, Hanson J, Goetz MP, Haddad TC, Iriarte D, Kaplan HG, Porter B, Rinn K, Thomas H, Thornton S, Tickman R, Varghis N; University of Alabama at Birmingham: Caterinichia V, Delos Santos J, Falkson C, Forero A, Krontiras H, Vaklavas C, Wei S; University of Arizona: Bauland A, Inclan L, Lewallen D, Powell A, Roney C, Schmidt K, Viscusi RK, Wright H; University of California, San Diego: Blair S, Boles S, Bykowski J, Datnow B, Densley L, Eghtedari M, Genna V, Hasteh F, Helsten T, Kormanik P, Ojeda-Fournier H, Onyeacholem I, Parker B, Podsada K, Schwab R, Wallace A, Yashar C; University of California, San Francisco: Alvarado MD, Au A, Balassanian R, Benz C, Buxton M, Chen YY, Chien J, D’Andrea C, Davis SE, Esserman L, Ewing C, Goga A, Hirst GL, Hwang M, Hylton N, Joe B, Lyandres J, Kadafour M, Krings G, Melisko M, Moasser M, Munter P, Ngo Z, Park J, Price E, Rugo H, van’t Veer L, Wong J, Yau C; University of Chicago: Abe H, Jaskowiak NT, Nanda R, Olopade F, Schacht DV; University of Colorado, Denver: Borges V, Colvin T, Diamond J, Elias AD, Finlayson C, Fisher C, Hardesty L, Kabos P, Kounalakis N, Mayordomo J, McSpadden T, Murphy C, Rabinovitch R, Sams S, Shagisultanova E; University of Kansas: Baccaray S, Khan Q; University of Minnesota: Beckwith H, Blaes A, Emory T, Haddad TC, Hui J, Klein M, Kuehn-Hajder J, Nelson M, Potter D, Tuttle T, Yee D, Zera R; University of Pennsylvania: Bayne L, Bradbury A, Clark A, DeMichele A, Domchek S, Fisher C, Fox K, Frazee D, Lackaye M, Matro J, McDonald E, Rosen M, Shah P, Tchou J, Volpe M; University of Texas, MD Anderson Cancer Center: Alvarez R, Barcenas C, Berry DA, Booser D, Brewster A, Brown P, Gonzalez-Angulo A, Ibrahim N, Karuturi M, Koenig K, Moulder S, Murray J, Murthy R, Pusztai L, Saigal B, Symmans WF, Tripathy D, Theriault R, Ueno N, Valero V; University of Southern California: Brown M, Carranza M, Flores Y, Lang J, Luna A, Perez N, Tripathy D, Watkins K; University of Texas Southwestern Medical Center: Armstrong S, Boyd C, Chen L, Clark V, Frankel A, Euhus DM, Froehlich T, Goudreau S, Haley B, Harker-Murray A, Klemow D, Leitch AM, Leon R, Li H, Morgan T, Qureshi N, Rao R, Reeves M, Rivers A, Sadeghi N, Seiler S, Staves B, Tagoe V, Thomas G, Tripathy D, Unni N, Weyandt S, Wooldridge R, Zuckerman J; University of Washington: Korde L, Griffin M, Butler B, Cundy A, Rubinstein L, Hixson C

## Electronic supplementary material

Supplementary Figure S1

Supplementary Figure S2

Supplementary Figure S3

Supplementary Figure S4

Supplementary Figure S5

Supplementary Figure S6

Supplemental Information
