# Supplementary figures and images for: DNA repair deficiency biomarkers and the 70-gene ultra-high risk signature as predictors of veliparib/carboplatin response in the I-SPY 2 breast cancer trial
Source: NPJ Breast Cancer. 2017 Aug 25;3:31. doi: 10.1038/s41523-017-0025-7 (PMC5572474; doi:10.1038/s41523-017-0025-7)

# BRCA1/2 germline mutation

wildtype

BRCA1/2+

VC

40

16

3

9

no pCR

pCR

Control

32

9

3

no pCR

pCR

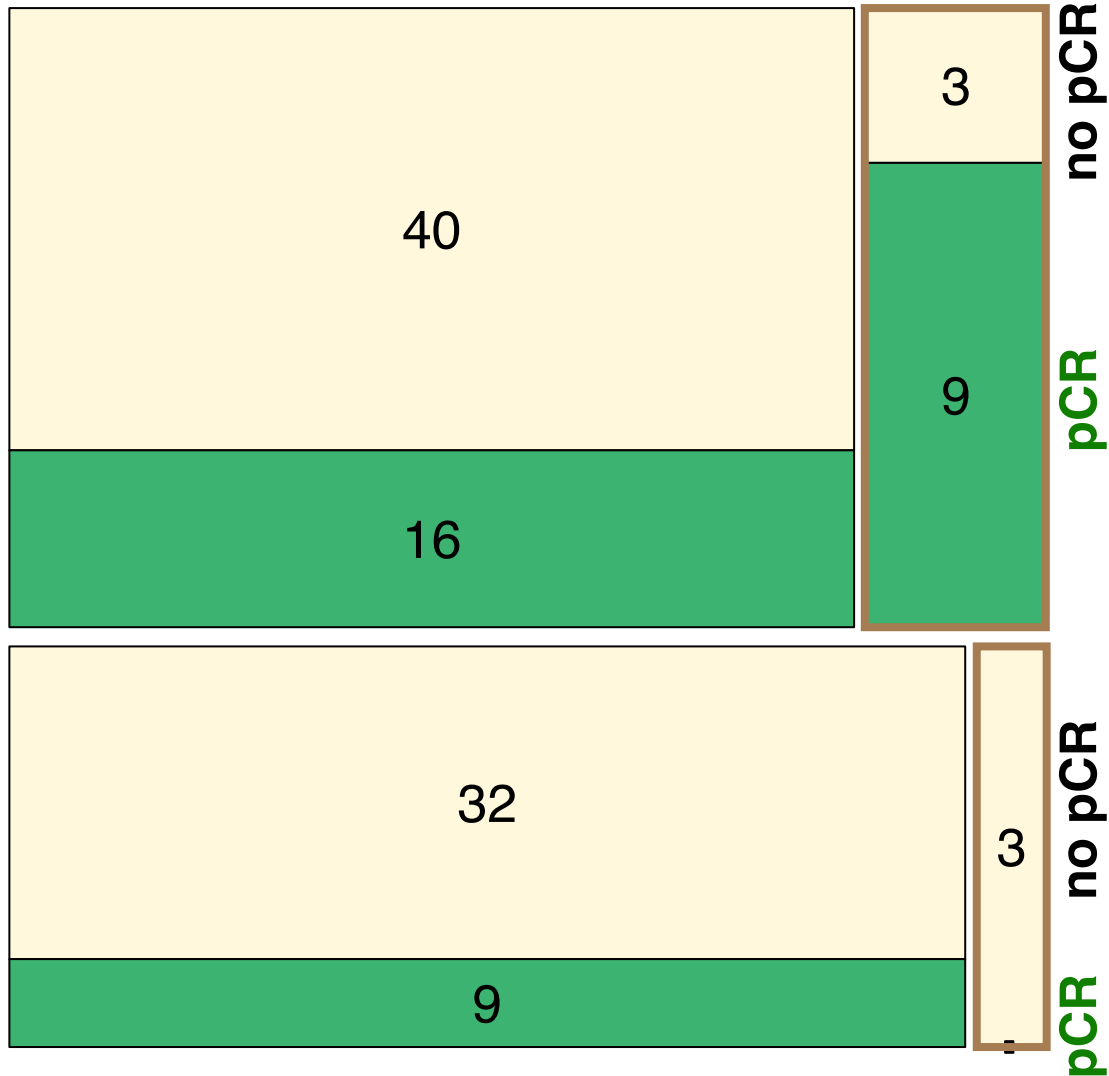

Supplement: Supplementary file 1 — Supplementary Figure S1 [file 41523_2017_25_MOESM1_ESM.pdf]

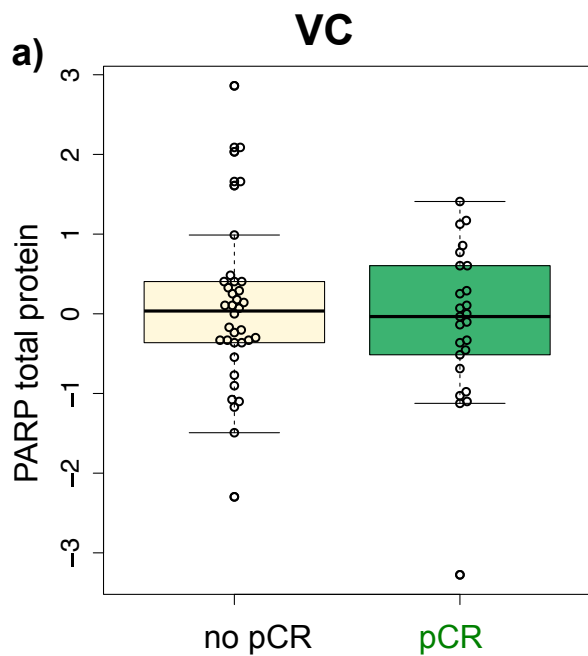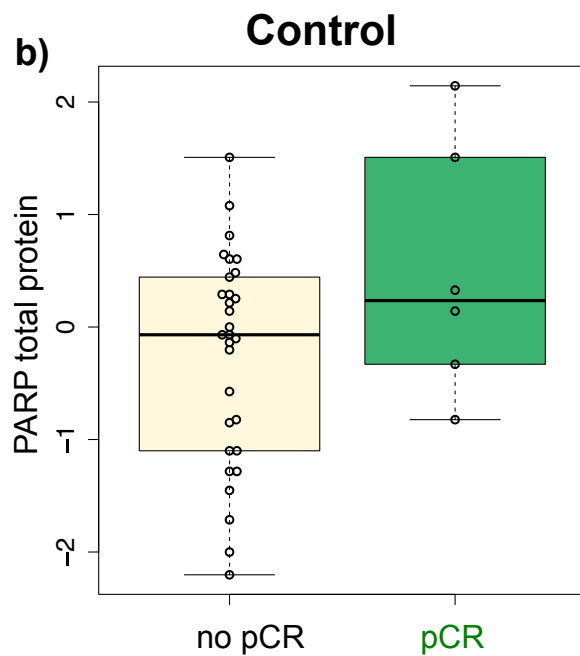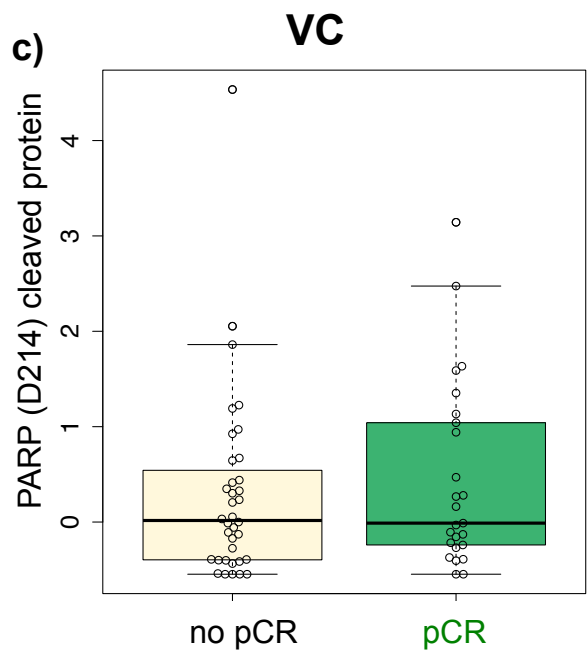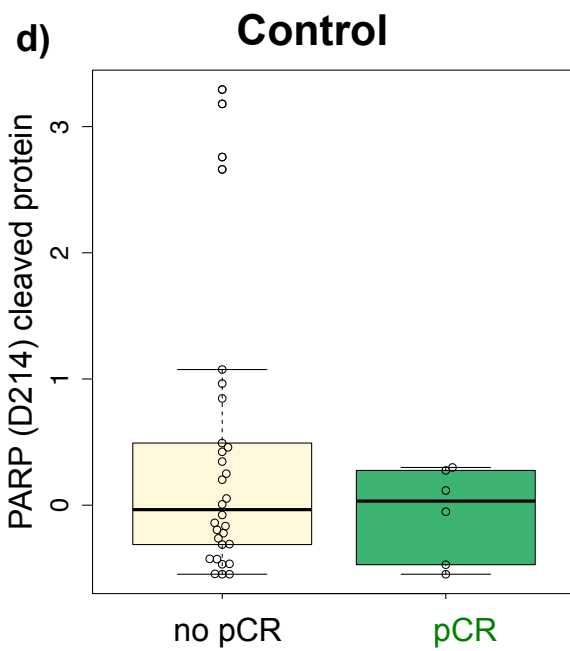

Supplement: Supplementary file 2 — Supplementary Figure S2 [file 41523_2017_25_MOESM2_ESM.pdf]

PARPi-7 Score

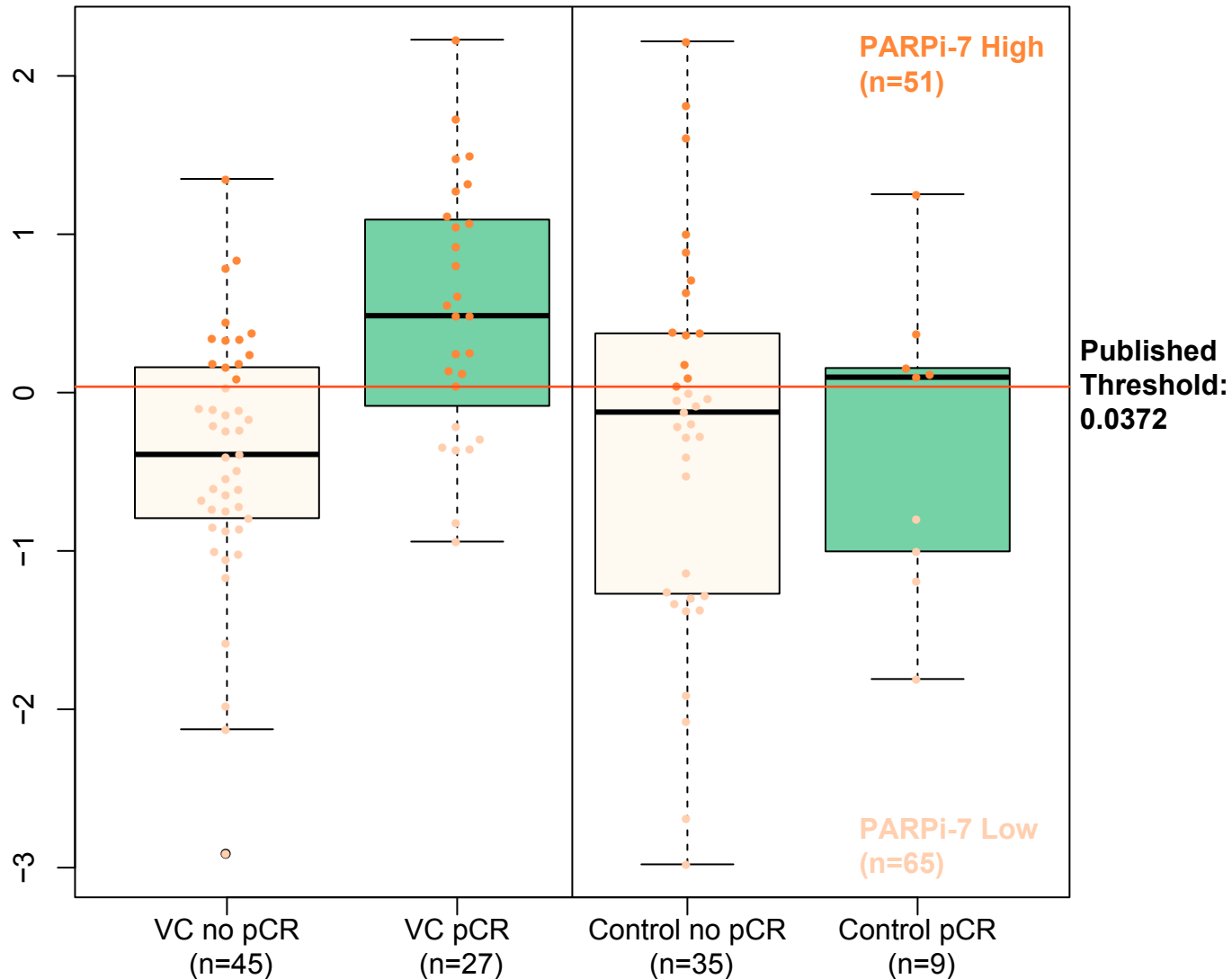

Supplement: Supplementary file 3 — Supplementary Figure S3 [file 41523_2017_25_MOESM3_ESM.pdf]
