# Supplementary material for: DNA repair deficiency biomarkers and the 70-gene ultra-high risk signature as predictors of veliparib/carboplatin response in the I-SPY 2 breast cancer trial
Source: NPJ Breast Cancer. 2017 Aug 25;3:31. doi: 10.1038/s41523-017-0025-7 (PMC5572474; doi:10.1038/s41523-017-0025-7)
Supplement: Supplementary file 4 — Supplementary Figure S4 [file 41523_2017_25_MOESM4_ESM.pdf]

a)

PARPi-7

	V/C (n=72)		Control (n=44)	
	PARPi-7 Low (n=39)	PARPi-7 High (n=33)	PARPi-7 Low (n=26)	PARPi-7 High (n=18)
TN (n=60)	5 / 15 (33%)	17 / 24 (71%)	1 / 7 (14%)	4 / 14 (29%)
HR+HER2- (n=56)	2 / 24 (8%)	3 / 9 (33%)	3 / 19 (11%)	1 / 4 (25%)

b)

*BRCA1*ness

	V/C (n=72)		Control (n=44)	
	Non-*BRCA1*ness (n=34)	*BRCA1*ness (n=38)	Non-*BRCA1*ness (n=27)	*BRCA1*ness (n=17)
TN (n=60)	5/ 7 (71%)	17/ 32 (53%)	2/ 6 (33%)	3/ 15 (20%)
HR+HER2- (n=56)	3/ 27 (11%)	2/ 6 (33%)	4/ 21 (19%)	0/ 2

c)

CIN70

	V/C (n=72)		Control (n=44)	
	CIN70 Low (n=53)	CIN70 High (n=19)	CIN70 Low (n=34)	CIN70 High (n=10)
TN (n=60)	14/ 25 (56%)	8/ 14 (57%)	3/ 14 (21%)	2/ 7 (29%)
HR+HER2- (n=56)	2/ 28 (7%)	3/ 5 (60%)	4/ 20 (20%)	0/ 3

d)

MP1/2

	V/C (n=72)		Control (n=44)	
	MP1 (n=32)	MP2 (n=40)	MP1 (n=34)	MP2 (n=10)
TN (n=60)	3 / 8 (38%)	19 / 31 (61%)	3 / 13 (23%)	2 / 8 (25%)
HR+HER2- (n=56)	1 / 24 (4%)	4 / 9 (44%)	4 / 21 (19%)	0 / 2
