# Supplementary material for: DNA repair deficiency biomarkers and the 70-gene ultra-high risk signature as predictors of veliparib/carboplatin response in the I-SPY 2 breast cancer trial
Source: NPJ Breast Cancer. 2017 Aug 25;3:31. doi: 10.1038/s41523-017-0025-7 (PMC5572474; doi:10.1038/s41523-017-0025-7)
Supplement: Supplementary file 5 — Supplementary Figure S5 [file 41523_2017_25_MOESM5_ESM.pdf]

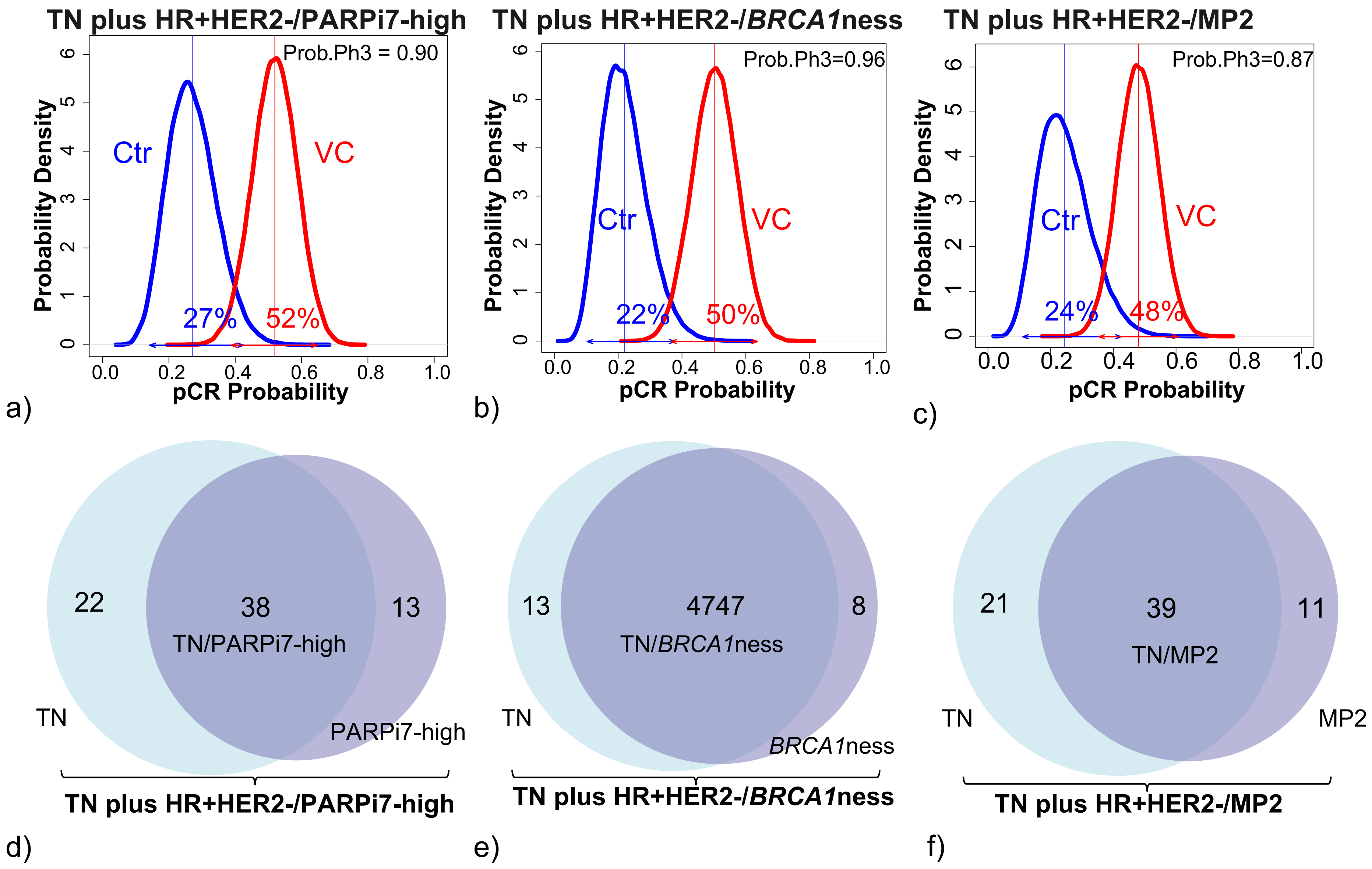
TN plus HR+HER2-/PARPi7-high
Prob.Ph3 = 0.90
Probability Density
Ctr
VC
27%
52%
pCR Probability
a)
TN plus HR+HER2-/*BRCA1*ness
Prob.Ph3=0.96
Probability Density
Ctr
VC
22%
50%
pCR Probability
b)
TN plus HR+HER2-/MP2
Prob.Ph3=0.87
Probability Density
Ctr
VC
24%
48%
pCR Probability
c)
22
38
TN/PARPi7-high
13
TN
PARPi7-high
TN plus HR+HER2-/PARPi7-high
d)
13
4747
TN/*BRCA1*ness
8
TN
*BRCA1*ness
TN plus HR+HER2-/*BRCA1*ness
e)
21
39
TN/MP2
11
TN
MP2
TN plus HR+HER2-/MP2
f)
