# Supplementary material for: DNA repair deficiency biomarkers and the 70-gene ultra-high risk signature as predictors of veliparib/carboplatin response in the I-SPY 2 breast cancer trial
Source: NPJ Breast Cancer. 2017 Aug 25;3:31. doi: 10.1038/s41523-017-0025-7 (PMC5572474; doi:10.1038/s41523-017-0025-7)
Supplement: Supplementary file 6 — Supplementary Figure S6 [file 41523_2017_25_MOESM6_ESM.pdf]

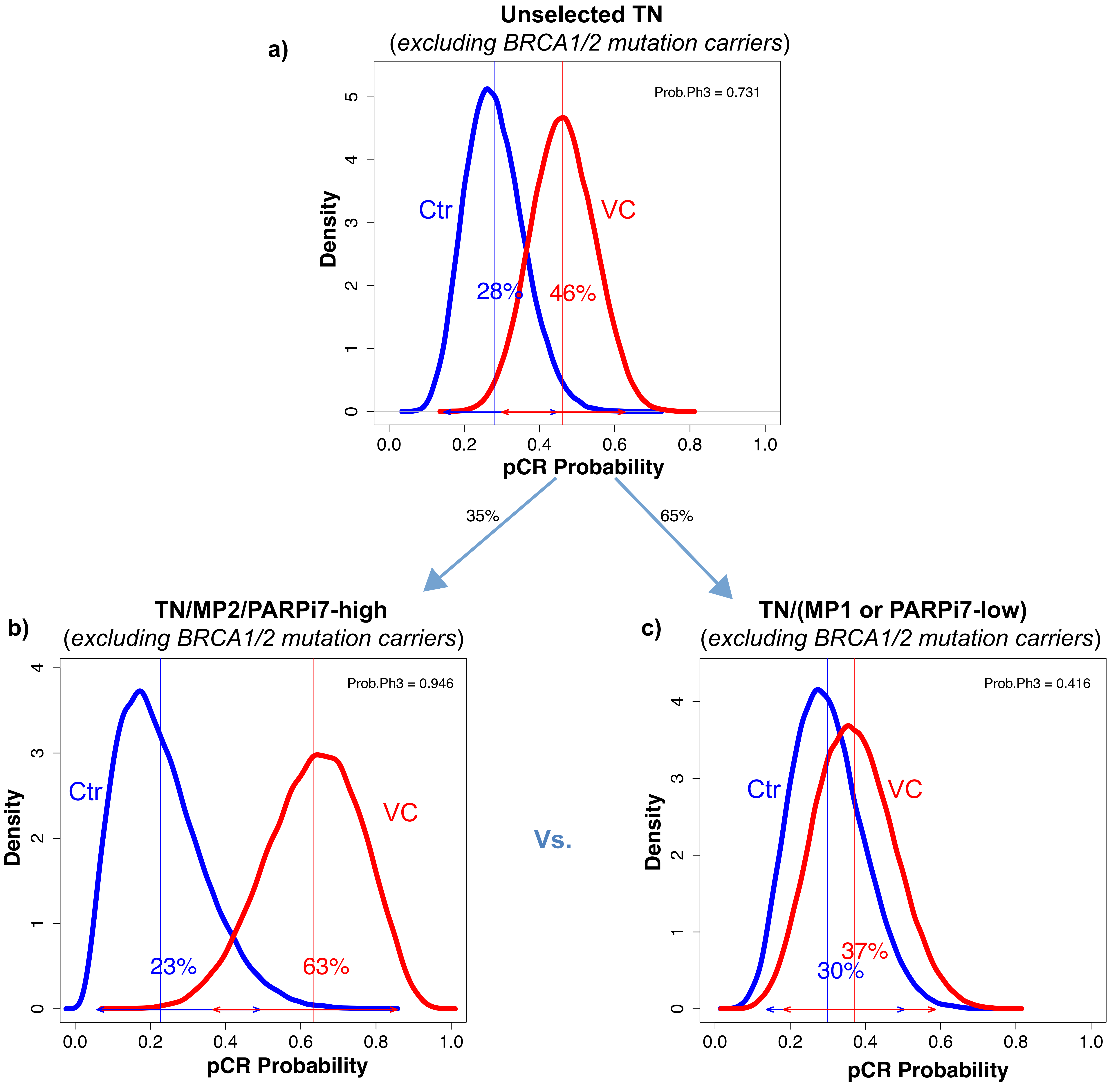

Unselected TN
(excluding BRCA1/2 mutation carriers)
a)
Prob.Ph3 = 0.731
Ctr
VC
28%
46%
Density
pCR Probability
0.0
0.2
0.4
0.6
0.8
1.0
0
1
2
3
4
5
35%
65%
TN/MP2/PARPi7-high
(excluding BRCA1/2 mutation carriers)
b)
Prob.Ph3 = 0.946
Ctr
VC
23%
63%
Density
pCR Probability
Vs.
TN/(MP1 or PARPi7-low)
(excluding BRCA1/2 mutation carriers)
c)
Prob.Ph3 = 0.416
Ctr
VC
37%
30%
Density
pCR Probability
