# Supplementary material for: DNA repair deficiency biomarkers and the 70-gene ultra-high risk signature as predictors of veliparib/carboplatin response in the I-SPY 2 breast cancer trial
Source: NPJ Breast Cancer. 2017 Aug 25;3:31. doi: 10.1038/s41523-017-0025-7 (PMC5572474; doi:10.1038/s41523-017-0025-7)
Supplement: Supplementary file 7 — Supplemental Information [file 41523_2017_25_MOESM7_ESM.pdf]

SUPPLEMENTARY INFORMATION for "DNA repair deficiency biomarkers and the 70-gene ultra-high risk signature as predictors of veliparib/carboplatin response in the I-SPY 2 breast cancer trial", by Wolf, Yau & van 't Veer, et al, npj Breast, 2017

A. SUPPLEMENTARY TABLES

Table S1. Table of response for BRCA1/2 germline mutation status, by receptor subtype and treatment arm.

	**VC (n=68)**		**Control (n=44)**	
	Wildtype (n=56)	BRCA1/2 mut+ (n=12)	Wildtype (n=41)	BRCA1/2 mut+ (n=3)
**TN (n=57)**	12/ 26 (46%)	8/ 10 (80%)	5/ 20 (25%)	0/ 1
**HR+HER2- (n=55)**	4/ 30 (13%)	1/ 2	4/ 21 (19%)	0/ 2

Table S2. Treatment effect assessments by Fisher's exact test in the expanded patient subsets composed of patients with TN breast cancer plus HR+HER2- patients who are biomarker-positive.

**Patient subset: graduating signature (TN) expanded by adding biomarker-positive patients**	**Treatment effect (pCR rate in VC vs. Ctr)**	
	OR	Fisher test: p-value
**TN**	4.04 [1.12-17.1]	0.028
**TN plus HR+HER2-/PARPi7-high**	3.38 [1.06-12.2]	0.026
**TN plus HR+HER2-/*BRCA1*ness**	4.03 [1.17-16.4]	0.019
**TN plus HR+HER2-/MP2**	4.17 [1.22-16.8]	0.012

Table S3. Table of Bayesian estimated pCR rates by treatment arm and predicted probability of phase 3 success in patient subsets defined by voting-method combined PARPi-7 and MP1/2 biomarkers, in the context of receptor subtype.

Biomarker subset	Combined VC sensitivity biomarker	Estimated pCR rate in VC [95% CI]	Estimated pCR rate in controls [95% CI]	Predictive probability of phase 3 success (300 pt)
**TN**				
**Unselected TN**	-	53% [38-67]	27% [13-43]	0.90
**TN/PARPi7-high/MP2 (42% of TN)**	TN/VC-Sensitive	75% [55-90]	23% [5.6-49]	0.99
**TN/(PARPi7-low and/or MP1) (58% of TN)**	TN/VC-Resistant	37% [19-55]	29% [13-48]	0.30
**HR+HER2-**				
**Unselected HR+HER2-**	-	15% [6-29]	19% [8-35]	0.11
**HR+HER2-/PARPi7-high/MP2 (11% of HR+/HER2-)**	HR+HER2-/VC-Sensitive	41% [14-71]	15% [3-40]	0.82
**HR+HER2-(PARPi7-low and/or MP1) (89% of HR+/HER2-)**	HR+HER2-/VC-Resistant	13% [4-25]	19% [7-35]	0.07

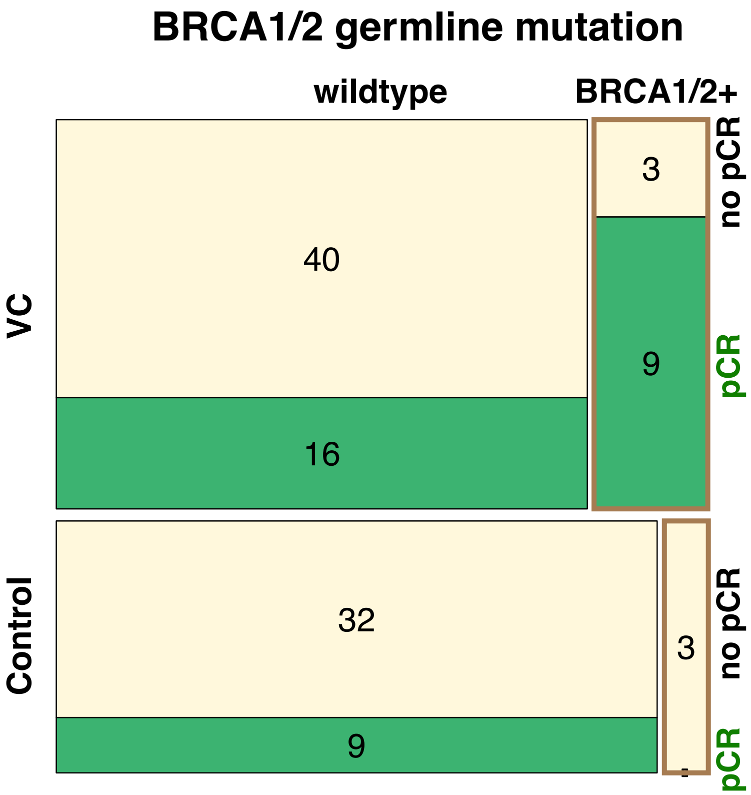

Figure S1. Mosaic plot showing response (pCR or no pCR) by BRCA1/2 germline mutation status, in each treatment arm.

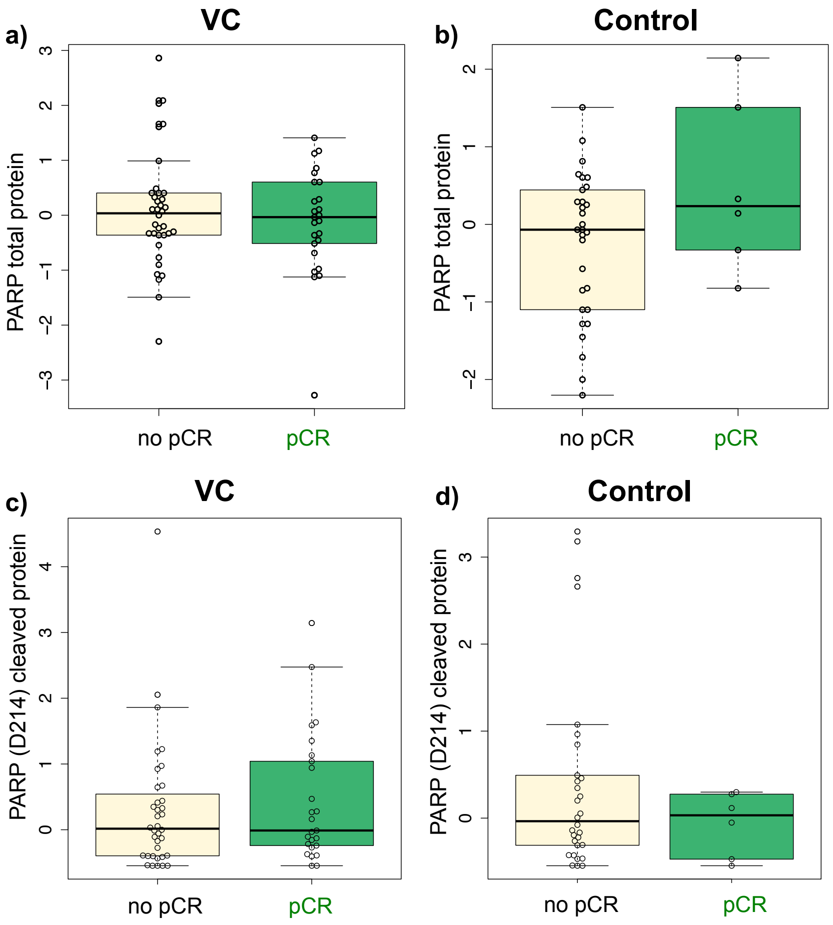

Figure S2. Box plots showing PARP and cleaved PARP protein levels by response within each treatment arm.

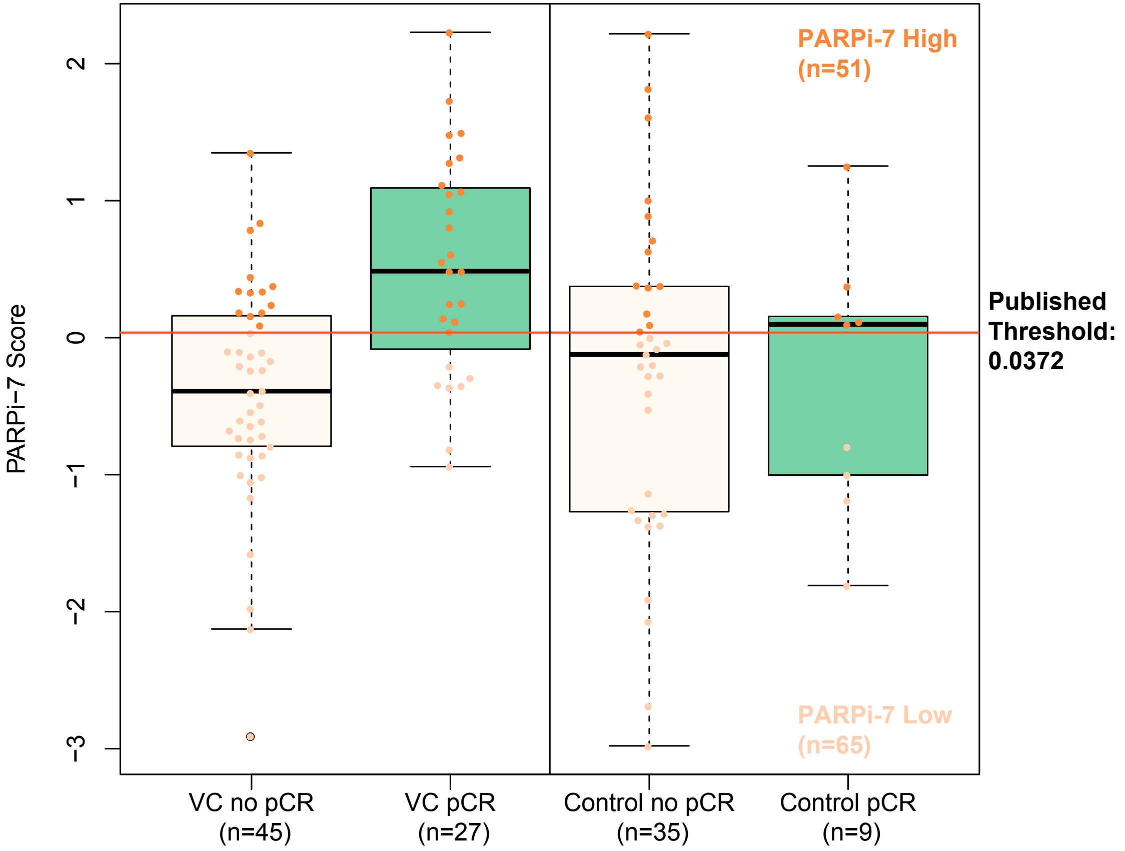

Figure S3. Box plots showing the continuous PARPi-7 score by response within each treatment arm. The dichotomizing threshold is denoted by a horizontal line at 0.0372, and by different colored dots representing patients classified as PARPi-7 High or PARPi-7 Low.

a)

PARPi-7

	V/C (n=72)		Control (n=44)	
	PARPi-7 Low (n=39)	PARPi-7 High (n=33)	PARPi-7 Low (n=26)	PARPi-7 High (n=18)
TN (n=60)	5 / 15 (33%)	17 / 24 (71%)	1 / 7 (14%)	4 / 14 (29%)
HR+HER2- (n=56)	2 / 24 (8%)	3 / 9 (33%)	3 / 19 (11%)	1 / 4 (25%)

b)

***BRCA1*ness**

	V/C (n=72)		Control (n=44)	
	Non-*BRCA1*ness (n=34)	*BRCA1*ness (n=38)	Non-*BRCA1*ness (n=27)	*BRCA1*ness (n=17)
TN (n=60)	5/ 7 (71%)	17/ 32 (53%)	2/ 6 (33%)	3/ 15 (20%)
HR+HER2- (n=56)	3/ 27 (11%)	2/ 6 (33%)	4/ 21 (19%)	0/ 2

c)

CIN70

	V/C (n=72)		Control (n=44)	
	CIN70 Low (n=53)	CIN70 High (n=19)	CIN70 Low (n=34)	CIN70 High (n=10)
TN (n=60)	14/ 25 (56%)	8/ 14 (57%)	3/ 14 (21%)	2/ 7 (29%)
HR+HER2- (n=56)	2/ 28 (7%)	3/ 5 (60%)	4/ 20 (20%)	0/ 3

d)

MP1/2

	V/C (n=72)		Control (n=44)	
	MP1 (n=32)	MP2 (n=40)	MP1 (n=34)	MP2 (n=10)
TN (n=60)	3 / 8 (38%)	19 / 31 (61%)	3 / 13 (23%)	2 / 8 (25%)
HR+HER2- (n=56)	1 / 24 (4%)	4 / 9 (44%)	4 / 21 (19%)	0 / 2

Figure S4. Response tables by receptor subtype, treatment arm, and biomarker, for the gene expression based dichotomous qualifying biomarker candidates (a) PARPi-7, (b) *BRCA1*ness, (c) CIN70, and (d) MP1/2.

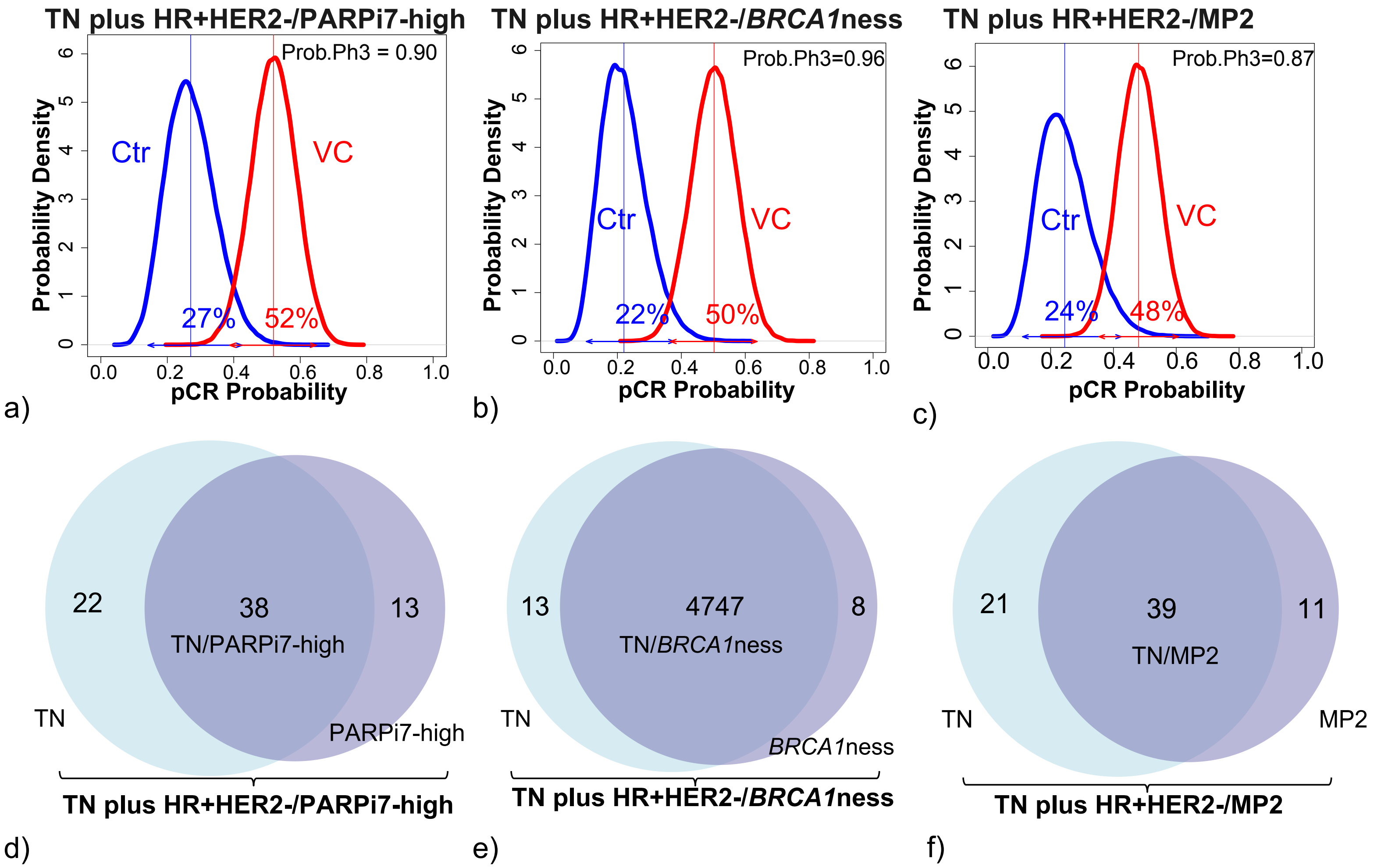

Figure S5. Expanding the 'predicted-sensitive' group beyond the graduating TN signature. To expand the triple negative signature, we added biomarker-positive HR+HER2- patients and assessed the a-c) Bayesian estimated pCR probability distributions by treatment arm and predicted probability of success in phase 3, for (a) TN plus HR+HER2-/PARPi7-high, (b) TN plus HR+HER2-/*BRCA1*ness, and (c) TN plus HR+HER2-/MP2 patients, respectively. d-f) Venn diagrams showing the overlap between TN and biomarker-positive subsets, for each signature, showing the increase in prevalence by including HR+HER2- biomarker-positive patients [(d) PARPi7-high; (e) *BRCA1*ness; and (f) MP2].

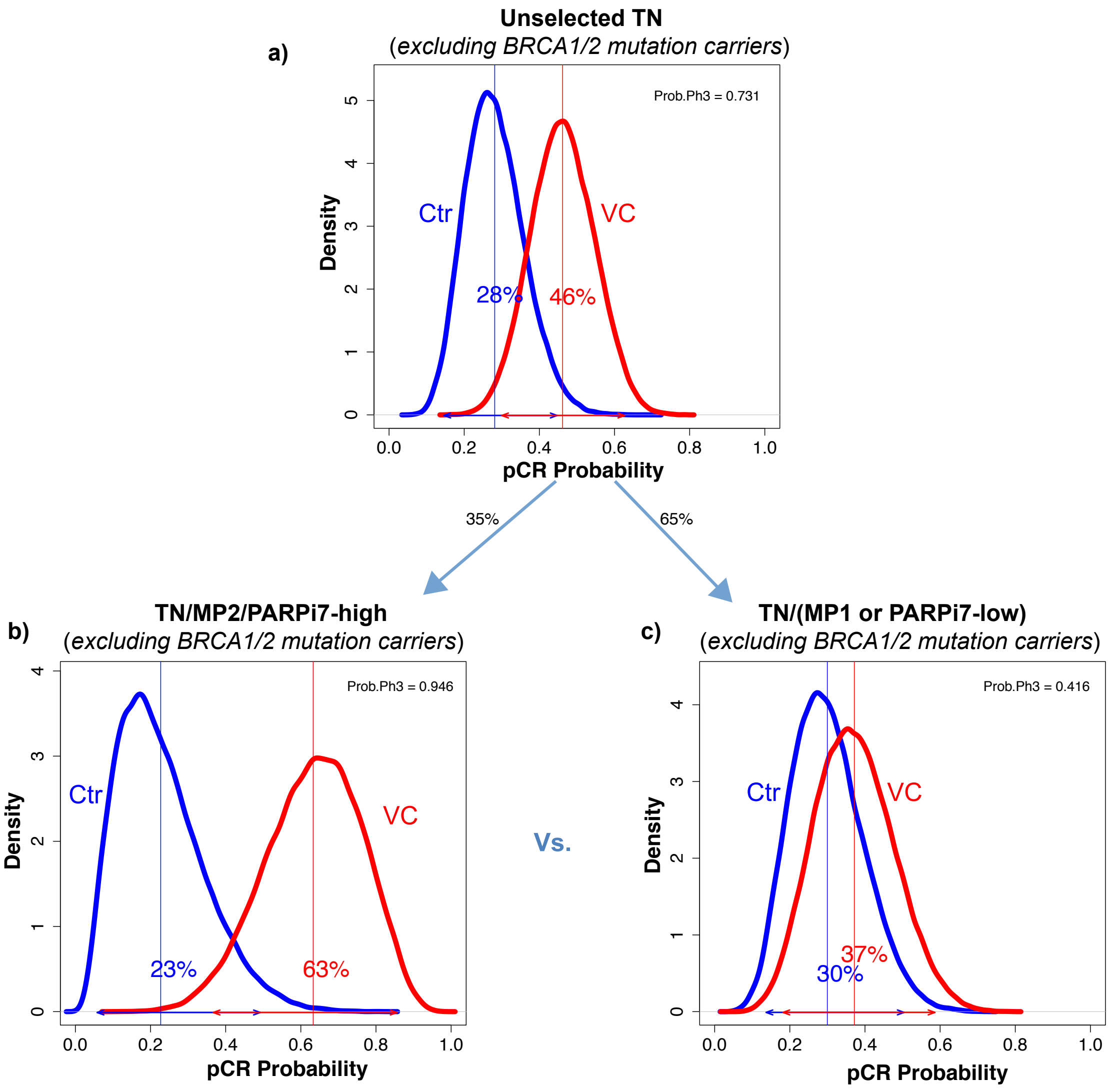

Figure S6. Combined VC-sensitivity markers in TN patients who do not carry a deleterious BRCA1/2 germline mutation. a-c) Bayesian estimated pCR probability distributions by treatment arm, for BRCA1/2-wildtype TN patients who are (a) unselected triple negative [TN], (b) predicted VC-sensitive (TN/MP2/PARPi7-high) and (h) predicted VC-resistant (TN/(MP1 or PARPi7-low)).

C. SUPPLEMENTARY METHODS

Patients and RNA profiling of pre-treatment tumor biopsies

Patients are eligible for the I-SPY 2 Trial if they have histologically confirmed invasive breast cancer greater than 2.5 cm that has been evaluated by Agendia as MammaPrint High Risk (of recurrence), or as low risk but HR- or HER2+, and are without evidence of distant metastatic disease. Figure 1a shows the study schema. Core needle biopsies of 16-gauge were taken from the primary breast tumor before treatment. Collected tissue samples are immediately frozen in Tissue-Tek® O.C.T.™ embedding media and then stored in -80°C until further processing. An 8µM section is stained with hematoxylin and eosin (H&E) and pathologic evaluation performed to confirm the tissue contains at least 30% tumor. A tissue sample meeting the 30% tumor requirement is further cryosectioned at 30 µM. Twenty to thirty sections are collected and emulsified in 0.5ml Qiazol solution and sent to Agendia, Inc., for RNA extraction and gene expression profiling on Agilent 44K microarrays. For each array, the green channel mean signal is log2-tranformed and centered within array to its 75th quantile as per the manufacturer's data processing recommendations. A fixed value of 9.5 is added to avoid negative values.

Biomarkers evaluated

BRCA1/BRCA2 germline mutation: BRCA1/2 mutation status was assessed using the CLIA-certified services provided by Myriad Genetics Laboratories for 70 patients treated with V/C and 44 concurrently randomized HER2- controls. Only patients with unambiguous interpretation of their germline mutation status were considered in the anaylsis. No variant detected or genetic variant, favor polymorphism were considered wildtype, whereas positive for deleterious mutation or suspected deleterious mutation in BRCA1 or BRCA2 were considered mutated. Variants of unknown significance were considered missing.

PARPi-7 signature: Agilent 44K expression data was pre-processed by quantile normalizing across the sample set. The PARPi-7 signature was evaluated as published [1], by 1) extracting probesets for predictor genes BRCA1, CHEK2, MAPKAPK2, MRE11A, NBN, TDG, and XPA and combining them into gene level summaries by averaging; 2) repeating (1) for normalization genes RPL24, ABI2, GGA1, E2F4, IPO8, CXXC1, and RPS10; 3) dividing each PARPi-7 predictor gene level by the geometric mean of the normalization genes; 4) log2-transforming each ratio and median centering across the population; and 5) calculating signature scores using the published weights and boundaries: Weights<-c(-0.5320, 0.5806, 0.0713, -0.1396, -0.1976, -0.3937, -0.2335), Boundaries<-c(-0.0153, -0.006, 0.0031, -0.0044, 0.0014, -0.0165, -0.0126), Score = Weights*(Genes -Boundaries). The score is then standardized to sd=1; and used for our qualifying biomarker evaluation. Patients are also dichotomized into PARPi-7 High and PARPi-7 Low classes using the in vitro derived threshold 0.0372. PARPi-7 High patients are predicted to be more DNA repair deficient and responsive to olaparib.

***BRCA1*ness signature**: The *BRCA1*ness classification is computed from Agilent full genome array data of the 77 signature genes using a nearest centroids model, as published [2,3]. Briefly, to develop the *BRCA1*ness classifier, 128 triple negative breast cancer samples were collected and gene expression data generated within the EU FP7 RATHER project. *BRCA1*ness classification was determined by multiplex ligation-dependent probe amplification (MLPA). The classification model of Diagonal Linear Discriminant Analysis (DLDA) with equal prior probabilities was run to select the 77 signature genes. The categorical signature was then translated to the diagnostic setting using a nearest centroids model. These centroids are used as the template for BRCA1-

like/sporadic-like, with a threshold selected to optimize performance in cross-validation analysis in RATHER. *BRCA1*ness patients are predicted to be more DNA damage repair deficient.

CIN70 signature: The chromosomal instability (CIN70) signature is evaluated as published [4,5] by extracting all available probesets for the 71 genes in the signature, collapsing them to gene level summaries by averaging across probesets, and calculating a continuous CIN70 score for each sample as the mean expression level of the gene set. Patients are classified as CIN70-high or CIN70-low/intermediate using the score demarcating the third and fourth quartiles as a threshold [4,5]. CIN70-High patients are predicted to have more chromosomal instability and thus DNA repair deficiency.

MP1/2: In I-SPY 2, patients were classified as MammaPrint High1 (MP1) or MammaPrint (ultra) High2 (MP2) by Agendia, Inc., using a pre-defined threshold applied to the MP 70-gene risk score evaluated on Agilent 44K arrays [6]. The threshold used is equivalent to the median cut-point of I-SPY 1 participants who fit the eligibility criteria for I-SPY 2 (-0.154 in the original I-SPY 1 dataset). Although MP1/2 is an established biomarker incorporated into the trial, MP2 is not an eligible signature for graduation due to the restricted enrolment of VC to HER2- patients.

PARP protein levels (RPPA): pre-treatment biopsy specimens were subjected to laser capture microdissection (LCM) to procure tumor epithelium for RPPA analysis, as published [7]. Approximately 10,000 cells are captured per sample. 2 biomarkers related to PARP1/2 signaling were evaluated by RPPA: total PARP1 protein (PARP.total) and PARP1 cleaved at D214 (PARP.D214).

Qualifying biomarker statistical analysis

We follow a pre-defined qualifying biomarker methodology for each individual proposed evidence-based biomarker approved by the I-SPY 2 Data access and Publication Committee (DAPC). Our Qualifying Biomarker Evaluation (QBE) methodology comprises a three stage filter designed to identify promising candidate biomarkers for validation in future trials (Figure 2b; [8]). The first stage follows the pre-specified analysis plan approved by the I-SPY 2 DAPC as documented in concept proposals. Briefly, in this analysis stage (Stage I), we assess the relative performance between arms (biomarker x treatment interaction, likelihood ratio $p < 0.05$) using a logistic model. If the biomarker x treatment interaction term coefficient is significant (LR $p<0.05$), and if the biomarker is significantly associated with response in the V/C arm ($p<0.05$), the biomarker succeeds as a qualifying biomarker (QB) and we proceed to the next stages of analysis, evaluation of the QB in the context of the graduating signature. As biomarker performance can be subtype-specific, all analyses are also performed adjusting for HR status as a covariate and within receptor subtypes, numbers permitting. Successful continuous QB's are dichotomized. In analysis Stage II, the QB-High group is added to the graduating (TN) signature to define a novel 'biomarker-positive' signature and the treatment effect in this group is evaluated using a logistic model. Given the small size of the trial, statistical calculations are descriptive (e.g. p-values are measures of distance with no inferential content). If the treatment effect is comparable to that of the graduating signature, and the prevalence is increased, we proceed to analysis Stage III. Here we modify the I-SPY 2 Bayesian model used in the trial's randomization engine to include the dichotomous QB to assess the novel signature by estimating pCR rates/distributions within the experimental and control arms of the trial, as well as the predictive probability of the V/C protocol demonstrating superiority to control in a 300-patient 1:1 randomized Phase III trial. In the Bayesian analysis, we analyze three novel 'biomarker-positive' signatures: QB-High patients; TN plus HR+/QB-High patients (expansion of graduating signature); and TN/QB-High patients (refinement of the graduating signature). This analysis does

not adjust for multiplicities. Analysis is performed using the R environment. Scripts are available upon request.

References

1 Daemen, A. *et al.* Cross-platform pathway-based analysis identifies markers of response to the PARP inhibitor olaparib. *Breast Cancer Res Treat*, doi:10.1007/s10549-012-2188-0 (2012).

2 Glas, A. *et al.* 532 Evaluation of a BRCAness signature as a predictive biomarker of response to veliparib/carboplatin plus standard neoadjuvant therapy in high-risk breast cancer: results from the I-SPY 2 trial. *European Journal of Cancer* **50**, 173, doi:10.1016/S0959-8049(14)70658-6.

3 Severson, T. M. *et al.* The BRCA1ness signature is associated significantly with response to PARP inhibitor treatment versus control in the I-SPY 2 randomized neoadjuvant setting. *Breast Cancer Res* (2017).

4 Carter, S. L., Eklund, A. C., Kohane, I. S., Harris, L. N. & Szallasi, Z. A signature of chromosomal instability inferred from gene expression profiles predicts clinical outcome in multiple human cancers. *Nat Genet* **38**, 1043-1048, doi:10.1038/ng1861 (2006).

5 Birkbak, N. J. *et al.* Paradoxical relationship between chromosomal instability and survival outcome in cancer. *Cancer Res* **71**, 3447-3452, doi:10.1158/0008-5472.CAN-10-3667 (2011).

6 Rugo, H. S. *et al.* Adaptive Randomization of Veliparib-Carboplatin Treatment in Breast Cancer. *N Engl J Med* **375**, 23-34, doi:10.1056/NEJMoa1513749 (2016).

7 Wulfkuhle, J. D. *et al.* Molecular analysis of HER2 signaling in human breast cancer by functional protein pathway activation mapping. *Clin Cancer Res* **18**, 6426-6435, doi:10.1158/1078-0432.CCR-12-0452 (2012).

8 Yau, C. *et al.* Abstract P3-06-37: I-SPY 2 qualifying biomarker evaluation (QBE): The challenge and opportunity for interrogating predicted pathways in an adaptive design biomarker rich trial. *Cancer Research 75(9 Supplement):P3-06-37; May 2015; DOI: 10.1158/1538-7445.SABCS14-P3-06-37*, doi:10.1158/1538-7445.SABCS14-P3-06-37.
